# Pilot of a Low-Cost Elementary School Handwashing Intervention in Bangladesh: Acceptability, Feasibility, and Potential for Sustainability

**DOI:** 10.4269/ajtmh.20-1335

**Published:** 2021-11-29

**Authors:** Farhana Sultana, Leanne Unicomb, Mahbubur Rahman, Shahjahan Ali, Dorothy L. Southern, Dalia Yeasmin, Rouha Anamika Sarkar, Kishor K. Das, Fosiul Alam Nizame, Peter J. Winch, Stephen P. Luby

**Affiliations:** ^1^ International Centre for Diarrhoeal Disease Research, Bangladesh (icddr,b), Dhaka, Bangladesh;; ^2^ Johns Hopkins Bloomberg School of Public Health, Baltimore, Maryland;; ^3^ Stanford University, Stanford, California

## Abstract

Schoolchildren frequently transmit respiratory and gastrointestinal infections because of dense person-to-person contact in schools. We piloted a low-cost handwashing intervention among elementary schoolchildren in Bangladesh. We trained teachers to lead behavior change communication sessions using flipcharts to encourage students’ handwashing before eating, after defecating, and after cleaning school toilets; provided handwashing stations (reservoirs with taps and stool + basin + soapy water solution [mix of 30 gm detergent with 1.5 L water] + pump top bottle with steel holder); and formed hygiene committees for maintenance and covering the recurrent cost of detergent. We evaluated intervention acceptability, feasibility, and potential for sustainability at 1 and 14 months after the intervention. At baseline, of 300 before eating events, no one washed hands with soap, and 99.7% (299) did not wash hands at all as soap was unavailable. Out of 269 after toileting events, 0.7% (2) washed hands with soap, and 88% (237) did not wash hands. After 4 weeks of the intervention, 45% (87/195 before eating events), 83% (155/186 after toileting events), and 100% (15/15 after cleaning toilet events) washed both hands with soapy water as children found it accessible, low cost, and child friendly. After 14 months, 9.4% (55/586 before eating events) and 37% (172/465 after toileting events) washed both hands with soapy water for health benefits. The intervention was acceptable and feasible; it overcame limited access to soap and water and was affordable as schools covered the recurrent costs of detergent. Further research should explore long-term habit adoption and impact on health and attendance.

## INTRODUCTION

Elementary school children frequently transmit respiratory and gastrointestinal infections because of dense person-to-person contact in schools and poor hygiene conditions.^1^^–^^3^ Handwashing with soap can prevent diarrhea and respiratory illness; improving school attendance and academic performance.^4^^–^^11^ Handwashing behavior change has been repeatedly promoted to prevent the spread of SARS-CoV-2 infection; frequent handwashing (every 2 hours) was one of the measures used to avoid school closures in China, Denmark, Norway, Singapore, and Taiwan.^12^

To prevent the spread of respiratory and gastrointestinal infections, the U.S. Centers for Disease Control and Prevention and WHO recommend handwashing at important key times including before eating, after using toilet following five to seven steps: 1) wet hands with clean and running water and apply soap, 2) lather hands by rubbing them together with the soap, and lather the backs of hands, between your fingers, and under the nails, 3) scrub hands for at least 20 seconds, 4) rinse hands well with clean and running water, and 5) dry your hands using a clean towel or air dry them.^13^^,^^14^

Components of school-based handwashing interventions include the provision of tippy taps, Super Jaboncin (a cartoon superhero who fights germs using water and soap), sanitizer, and soapy water have been widely tested in low- and middle-income countries.^15^^–^^21^ Tippy taps provided a low-cost enabling technology, and increased handwashing rates,^15^^,^^16^ the use of sanitizer resulted reduced absenteeism,^18^^,^^22^ and the provision of soapy water reduced bar soap theft.^20^^,^^23^

However, improving handwashing behaviors in schools in low- and lower-middle-income countries, including Bangladesh, has proven difficult to sustain as soap is expensive, schools lack funding and infrastructure, and soap placed at common handwashing stations may be stolen.^24^^–^^26^ Previous studies found the hands of elementary school children were less clean compared with older children in secondary schools in Bangladesh (27% versus 49%, *P* < 0.000).^26^^–^^28^

The National Standards for Water, Sanitation and Hygiene for Schools in Bangladesh 2011, the National Hygiene Promotion Strategy 2012, and the Ministry of Education Statement 2015 all aimed to increase handwashing in schools by providing soap and running water, improving handwashing behavior, operationalizing cleaning and maintenance routines, and creating school-based funding mechanisms.^29^^,^^30^ However, a weak and complicated budgetary allocation and procurement process in Bangladeshi schools fails to ensure an operating budget for basic supplies, including soap.^31^^,^^32^ Most of the studies on handwashing behavior change are short-term and do not assess the prolonged habit adoption after the intervention period,^33^ though this is a key consideration for cost-effectiveness and sustainability. We developed and pilot-tested a low-cost handwashing intervention to provide practical infrastructure, affordable supplies, and a pathway to institutionalize improved hygiene practices. We also collected long-term postintervention follow-up data on handwashing uptake to assess prolonged and sustained habit adoption. This article describes school children’s baseline knowledge, practices, perceptions, motivations, and barriers of hand hygiene, and the effectiveness, acceptability, feasibility, and potential for sustainability of a school handwashing intervention.

## MATERIALS AND METHODS

### Study setting and study participants.

The essential elements of the study have been described previously.^26^ Briefly, we conducted this study among four urban and four rural elementary school students (both government and nongovernment) in Bangladesh from May 2011 to September 2013. This was a proof-of-concept study that we conducted in multiple phases (Supplemental Table 1) as described below in more detail.

### Phase 1: Formative study of current knowledge, practices, perceptions, motivations, and barriers.

Out of eight schools, two urban and two rural schools participated in the formative study from September to November 2011. The purpose of the formative study was to identify and develop an intervention that is feasible, acceptable with the potential to be sustainable at improving handwashing with soap. We explored student-friendly technological options for handwashing stations, the most effective participatory implementation process with the best suited channel(s) of communication and operationalize and maintenance methods to make consistent the use of the preferred set of interventions.

#### Spot checks of facilities.

Fieldworkers conducted spot checks of baseline water, sanitation, and hand hygiene facilities (Supplemental Appendix 1a and 1b) in each of the four schools to explore the factors in the physical environment favoring or discouraging handwashing with soap before eating and after toileting or after a respiratory related condition (Table 3).

#### Structured observations.

Fieldworkers conducted a 4-hour long structured observation on three consecutive days to explore students’ baseline hand hygiene practices. We prepared a standard hand hygiene behavior assessment tool to observe and record hand hygiene practices of each student. Fieldworkers informed school administrators, but not individual students, about the purpose of the visit. They placed themselves in a convenient place at each school compound and noted any hand hygiene behavior related events on the pre-coded structured observation form.

#### Baseline student survey.

A previous study conducted by UNICEF found 10% of Bangladeshi schoolchildren washed hands after defecation at baseline and 35% after handwashing intervention. A sample of 102 students would provide 80% power and 95% confidence with design effect of 2 to account for clustering. We increased the targeted number of students to 200 to provide greater power given clustering by school and class. We randomly selected 50 students from each school. The schools averaged 475 students. Fieldworkers prepared a list of all students from grades 4 and 5 using class registers and then randomly drew 25 names from an envelope from each grade.

#### Qualitative interviews.

We purposively selected participants for in-depth interviews and focus group discussions based on the objective of the study, students’ enrolment in grades 4 and 5, and availability and willingness to participate. Fieldworkers conducted 16 in-depth interviews and 12 focus group discussions with the head, science and assistant teachers, school management committee and parent-teacher association members, janitors, male and female students, and female students to gather information related to perceptions on hygiene-related topics, suggestions for the design and content of the set of interventions, and the process of implementation. We crosschecked findings from different methods to increase validity.

#### Participatory exercises.

Fieldworkers conducted 12 drawing, vignette, ranking, and puzzle exercises with purposively selected students to explore and identify what attributes they wanted in an ideal handwashing station and their perception of symptoms and signs of diarrhea. These participatory techniques were designed to be child friendly, nonthreatening, and facilitating the expression of views by the child, without adults being involved.^34^^,^^35^ For example, we explored the use of artwork/drawings with the children to help them express their ideas more easily and to investigate what attributes they want in an ideal toilet. We applied vignette approaches to explore their perception of symptoms and signs of diarrhea and respiratory diseases and the routes of transmission and understand social norms related to these diseases. We provided the cards depicting a variety of symptoms and asked to match the symptoms to the illnesses. To understand the children’s preferred option for handwashing stations and motivators, we used ranking and puzzle exercise by displaying colorful pictures and cartoon illustrations. During the discussions, we presented options stating the advantages and disadvantages in light of their environmental context. Through the discussion, we obtained an idea on the preferred intervention option by asking each child to place a colored stone on the most preferred option for each of our category of options that they would like to see in their school grounds. At the end of the discussion, the number of stones was counted in each category and the reasons for their choices discussed.

#### Intervention development and trial of improved practices.

We organized an intervention development workshop with teachers, school management committee members, and coauthors (F. S., L. U., F. A. N., S. A., and P. W.) to seek feedback on a feasible handwashing intervention.

Based on the findings from Phase1, we developed an on-site school intervention that included 1) training teachers to lead behavior change communication sessions using 12 × 17-inch pictorial flipcharts and cue cards (Supplemental Figure 1); 2) provision of two handwashing stations: bucket with taps + 10 L bucket as basin + soapy water bottle containing a low-cost solution of 30 gm detergent powder mixed with water in a 1.5-L pump top bottle + steel holder (Supplemental Table 2); and 3) formation of a hygiene committee of students, teachers, janitors, parents, and school management committee members to maintain the handwashing stations, pay for the detergent cost, promote regular hand hygiene practices, and overcome the weak accountability for allocation of budget line items to essential supplies.

To train the schoolteachers, we invited them to International Center for Diarrheal Disease Research, Bangladesh (icddr,b) and held a1-day training on how to conduct sessions, deliver messages using flipcharts and maintain the handwashing stations. This training of teachers aimed to create a resource person in schools to promote cough etiquette practices, to deliver communication sessions, and to ensure ownership of the project for long-term sustainability of cough etiquette behavior change.

The flipcharts entailed detailed discussion on topics related to 1) importance of healthy life and regular school attendance, 2) impact of unhygienic practices on health and school attendance, 3) route of disease transmission, 4) healthy hands, 5) barriers to handwashing with soap in school, 6) introduction of the handwashing stations, 7) soapy water preparation method, 8) important key times/antecedents of handwashing, 9) handwashing steps adapted from CDC and WHO, and 10) maintenance of the handwashing stations. The cue card identified important key times behavioral antecedents for handwashing.

The purpose of the hygiene committee was to ensure ownership/leadership of the project for long-term sustainability of handwashing behavior change, and to help the school community decide on implementation process, and on methods to fund the recurrent cost of soap, cleaning materials, and associated maintenance cost. The committee consisted of 18 members: 10 students of grade 4 and 5 (as members to monitor and support the maintenance of the handwashing stations), one head and two assistant teachers (as secretary and assistant secretary to conduct sessions and ensure funds), two education officials (as advisor and coadvisor to monitor and guide the intervention activities), one janitor (as supportive staff to maintain the handwashing stations), and one school management committee and one parent–teacher association members (as chair and cochair to support funds) in each school.

We then conducted a 2-week trial of improved practices,^36^ to pretest the intervention materials in each four of the baseline formative schools. We then conducted structured interviews with eight teachers and 100 students to explore the appropriateness, feasibility, and acceptability of these interventions in the school communities.

### Phase 2: Piloting the intervention for acceptability and feasibility.

We piloted the intervention in the remaining four schools in June 2012. The purpose of the pilot phase was to evaluate and assess the acceptability and feasibility of the intervention in terms of uptake of target practices through the outcomes of increased knowledge of key times to wash hands with soap, perceived barriers of handwashing and perceived subjective and descriptive norms for handwashing.

#### Spot checks of facilities.

Fieldworkers conducted spot checks of handwashing facilities (Supplemental Appendix 1a and 1b) before and after intervention rollout to explore if the improvement in the physical environment (provision of handwashing stations) favoring or discouraging handwashing with soapy before eating, and after toileting and after cleaning toilet (Table 3). We demonstrated the use of soapy water and handwashing stations, forming the hygiene committee, and training teachers to lead, deliver, and continue behavior change communication sessions in conjunction with the existing weekly hygiene classes. We assessed the intervention 4 weeks after the commencement of the sessions in July 2012.

#### Structured observations.

Fieldworkers monitored the intervention for 1 month by conducting a 4-hour long structured observation on six occasions (days 1, 2, 3, 7, 14, and 30) after the first class on hand hygiene commenced.

#### Qualitative assessment.

Fieldworkers conducted qualitative interviews to explore the acceptability and feasibility of the intervention. We purposively selected a variety of stakeholders and students based on their availability and willingness to participate. Fieldworkers conducted two in-depth interviews with the janitors at urban schools and 14 focus group discussions (four each with students of grade 4 and 5 who were observed to wash hands, with those who were observed not to wash hands, teachers, school management committee, and parent–teacher association members, and two with the student members of hygiene committee at rural schools.

### Phase 3: Follow-up assessment at 14 months to explore sustainability.

The purpose of this phase was to explore the potential for sustainability of the intervention through understanding variations in uptake and practices and determining facilitating and inhibiting factors for handwashing and the various intervention components are perceived by the school community. Fieldworkers revisited pilot intervention schools in August 2013, 14 months after the intervention commenced, and 13 months after the study staff withdrew.

#### Spot checks of facilities.

Fieldworkers conducted four spot checks of facilities (Supplemental Appendix 1a and 1b) in each of the four schools to explore if the handwashing stations that we provided during the pilot testing phase of the study were in use and maintained by the hygiene committee (Table 3).

#### Structured observations.

They also conducted 4-hour structured observations of students using a checklist for three consecutive days to identify students who washed or did not wash hands.

#### Focus group discussions.

Fieldworkers conducted 12 focus group discussions with the purposively selected students of grades 4 and 5 who washed and did not wash hands, and with the teachers, school management committee, and parent–teacher association members to understand their perceptions, perceived benefits, and barriers of handwashing with soapy water.

### Data collection and analysis.

We collected both qualitative and quantitative data using a baseline survey, follow-up observations of uptake, and qualitative assessments.

All the observations were made on the school premises, and the interviews were conducted in Bengali in classrooms during school hours and lunch breaks. We structured our study questionnaires and findings using the integrated behavioral model for water, sanitation, and hygiene (IBM-WASH) framework adapted for school WASH interventions^37^ to determine the influential behavioral factors (Table 1). Detailed analysis using different factors of this model in this study have been described previously.^26^ Briefly, the model describes 1) contextual dimension–the social and physical environment in which the WASH behaviors and technologies are implemented; 2) a psychosocial “software” dimension—social and psychological factors that affect WASH practices; and 3) a technological “hardware” dimension factors affecting adoption of WASH technologies. In this study, the contextual dimension represents the formative study findings that we used to develop the intervention package. The psychosocial (e.g., the behavior change communication strategies) and technological (e.g., handwashing stations) dimension represents the findings after the intervention was implemented (Table 1).

**Table 1:** Conceptual framework: Analysis of contextual, psychosocial, and technological dimensions of the IBM-WASH^37^ based on the baseline, pilot, and follow-up study findings in urban Dhaka and rural Mymensingh, Bangladesh, 2012–2013

Levels	Contextual	Psychosocial “Software”	Technology “Hardware”
School system/Structural	Schools lack designated fund to purchase soap, and regulations to promote and support handwashing with soap both in urban and rural schools. School used their limited contingency fund for teachers to cover the recurrent cost of soapy water.	Communication sessions were merged with schools’ weekly/biweekly regular hygiene sessions.	Teachers and management committee members of the hygiene committee covered the recurrent cost detergent (US$ 0.04 per day for an average of 250 students) from school’s contingency funds, and from their own pockets sometimes. The hardware was also low-cost (US$ 10.6) and locally available that can potentially be replaced, sustainable and scaled out for future interventions.
School/school principal	Piped water supply in urban schools and hand pumps in rural schools Both schools lacked water in summer. Urban school had responsible janitors to maintain WASH facilities that rural schools lacked.	Formation of hygiene committees created school ownership that led toward a shared goal of handwashing promotion and practices at school compounds, as well as in the family and community. Younger students of grade III also took the lead to maintain handwashing stations after the older students of grade V finished their study and left school.	Handwashing stations were installed near to the toilets at school premises. Two handwashing stations served for the separate and gender-based use of male and female students. Janitors at urban and older students at rural schools stored water in the buckets, prepared soapy water and maintained the cleanliness.
Interpersonal/classmates and teachers	Experienced and skilled teachers to promote hand hygiene though inadequate number of teachers resulted overload in work.	Students, teachers, and management committee members disseminated hand hygiene intervention messages to the community.	Older children (grade III, IV and V) demonstrated handwashing behavior and the use of the handwashing station and soapy water to the younger children (grade I and II).
Individual	Both urban and rural elementary school represented low socioeconomic status	Older students comparatively had more self-efficacy to wash hands. Students improved their knowledge about handwashing at different key time after the weekly/biweekly hygiene sessions using flipcharts	Soapy water perceived as a low-cost alternative solution to bar soap that reduced barriers related to theft, misuse, and recurrent costs. Therefore, both urban and rural schools covered the cost of detergent even after the project ended. The handwashing station with bucket, basin and stool perceived as a complete technology for handwashing. The station was child friendly with attractive red-colored bucket and pump-top soapy water bottle. Especially rural children liked pump top bottle compared with pumping tube well for handwashing which was difficult.
Behavioral/habitual	Schools break for half an hour during which rural school (11.30–12 pm) children go to home (as homes are in close distance) for food, and urban school (10.30–11 am) children either eat in the classrooms (food from home) or buy snacks from nearby shops.	Because of the lack of bar soap supply, school children do not wash hands before eating. Children had perceived health benefits for handwashing, though girls reported more concerns about disgust and cleanliness than boys.	Rural schools lack school boundary, therefore, they had to take the handwashing stations to the office rooms daily to avoid theft problems after school hours.

IBM-WASH = Integrated Behavioral Model for Water, Sanitation and Hygiene.

For quantitative data analysis, we performed a descriptive summary of socio-demographic characteristics, and reported student survey data that included investigating the IBM-WASH model (knowledge, practice, beliefs, subjective and descriptive norms, access to facility and self-efficacy of promoted hygiene behaviors).We compared the proportion of observed hand hygiene practices before eating, after toileting and after cleaning school toilet at baseline, pilot, and 14 months follow-up using cluster adjusted Pearson’s χ^2^ test.^38^

Trained fieldworkers transcribed the audiorecorded focus group discussion and in-depth interview data and then translated it into English. We prepared the interview guideline based on research objectives and conducted a thematic content analysis to provide descriptive and systematic results. We analyzed each in-depth interview and each focus group discussion separately; however, we have drawn inferences collectively from both types of data and presented these in the results.

### Ethics.

We obtained permission from the Government of Bangladesh Divisional Primary Education Office, Dhaka, to work in specific schools in Dhaka and Mymensingh districts for research purposes. All teachers provided written consent, and students assented to participate before we collected data from them. The icddr,b Ethical Review Committee reviewed and approved the study protocol.

## RESULTS

### School participants.

Most study respondents were students of grades 4 and 5 with a mean age of 10.1 years old (SD 1.3) (Table 2).

**Table 2:** Socio demographic characteristics of study respondents in urban Dhaka and rural Mymensingh, Bangladesh, 2012–2013

Indicators	Baseline study	Pilot testing of intervention	14-month follow-up assessment
	*n* (%)	*n* (%)	*n* (%)
Respondent characteristics			
Student	248 (89)	144 (86)	24 (50)
Female	139 (56)	77 (53)	12 (50)
Adults	30 (11)	24 (14)	24 (50)
Female	07 (23)	08 (33)	08 (33)
Mean age (years) of respondent			
Student	11	11	10
Adults	41	38	40
Education of the respondent			
Grade III	0	2 (1)	0
Grade IV	124 (45)	82 (48)	14 (29)
Grade V	124 (44)	62 (37)	12 (25)
Elementary	6 (2)	6 (4)	4 (8.3)
Secondary	9 (3)	0	3 (6.2)
Tertiary	7 (3)	8 (5)	5 (10)
Graduation	8 (3)	8 (5)	10 (21)
Occupation of the guardian of the students			
Farmer	49/248 (20)	17/ 144 (12)	5/24 (21)
Salaried Gov. job	39/248 (16)	20/ 144 (14)	5/24 (21)
Small business	68/248 (27)	71/144 (49)	7/24 (29)
Nonagricultural labor/Rickshaw puller	50/248 (20)	20/144 (14)	5/24 (21)
Other*	42/248 (17)	16/144 (44)	2/24 (8)

* Day labor, driver, mason, mechanic carpenter, living abroad, gas contractor and bus helper.

### Phase 1: Formative study of knowledge, practices, perceptions, motivations, and barriers.

Spot checks of facilities showed that only one urban school had a separate handwashing station; this station had intermittent piped water into a broken basin next to the toilet. For both rural schools, the area surrounding the outdoor tube well areas served as the handwashing stations. Soap was not present in any of the four schools (Table 3).

**Table 3:** Schools physical environment and spot checks of handwashing facilities at baseline, pilot, and follow-up in elementary schools in urban Dhaka and rural Mymensingh, Bangladesh, 2012–2013

	Urban government school		Urban registered nongovernment school		Rural government school		Rural registered Nongovernment school	
Indicators	Formative	Pilot	Formative	Pilot	Formative	Pilot	Formative	Pilot
Schools’ physical environment and handwashing facilities at baseline								
Total number of students								
200–500	0	0	1	0	1	1	1	1
600–1100	1	1	0	1	0	0	0	0
Total number of toilets	2	4	1	1	1	1	1	1
Source of water for handwashing								
Piped water supply	1	1	0	0	0	0	0	0
Deep tube well	0	0	1	1	0	0	0	0
Shallow tube well	0	0	0	0	1	1	1	1
Type of handwashing facility								
Basin	1	0	0	0	0	0	0	0
Toilet area	0	1	1	1				
Tube well area	0	0	0	0	1	1	1	1
Water at the handwashing station	0	1	0	0	1	1	0	0
Soap/hand cleansing material at the handwashing station	0	0	0	0	0	0	0	0
Handwashing facilities after the intervention	Pilot	Follow-up	Pilot	Follow-up	Pilot	Follow-up	Pilot	Follow-up
Availability of handwashing facilities/intervention materials								
Water	1	0	1	1	1	1	1	1
Soapy water	1	0	1	1	1	1	1	1
Detergent powder	1	0	1	0	1	1	1	1
Metal holder	1	0	1	1	1	1	1	1
Flipcharts	1	1	1	1	1	1	1	1
Cue cards	1	1†	1	1	1	1†	1	1‡
Functionality of the handwashing stations	1	1	1	1	1	1	1	1
Weekly hygiene sessions continued	1	0‡	1	0‡	1	1	1	1

Yes = 1, No = 0.

† Some parts were missing.

‡ Ceased 7–12 months after the intervention.

During the structured observations, fieldworkers observed 569 student opportunities for handwashing: 53% (300) before eating and 47% (269) after toileting. In the 300 handwashing opportunities before eating, none of the children washed hands with soap, and 99.7% (299) did not wash hands at all. Among the 269 handwashing opportunities after toileting, 0.7% (2) children washed hands with soap, and 88% (237) did not wash hands at all (Table 4).

**Table 4:** Proportion of observed hand hygiene practices before eating, after toileting, and after cleaning toilet at baseline, pilot, and follow-up in urban Dhaka and rural Mymensingh, Bangladesh, 2012–2013

Indicators	Baseline formative study	Pilot testing of the intervention	14-month follow-up assessment
Before eating	*N* = 300	*N* = 195	*N* = 586
Washing hands using water only % (*n*)	0.3 (1)	0	0.9 (5)
Washing both hands using soap/soapy water % (*n*)	0.0 (0)	45 (87)*	9 (55)*
Ate with spoon % (*n*)	0	0	1 (6)
Did not wash hands at all % (*n*)	99.7 (299)	55 (108)*	89 (520)
After toileting	*N* = 269	*N* = 186	*N* = 465
Washing hands using water only % (*n*)	11.2 (30)	0	25 (118)*†
Washing both hands using soap/soapy water % (*n*)	0.7 (2)	83 (155)*	37 (172)*
Did not wash hands at all % (*n*)	88 (237)	17 (31)*	38 (175)
After cleaning toilet‡		*N* = 15	*N* = 4
Washing hands using water only % (*n*)	0	0	0
Washing both hands using soap/soapy water % (*n*)	0	100 (15)	50 (2)
Did not wash hands at all % (*n*)	0	0 (15)	50 (2)

* Proportion is significantly different compared with baseline formative study using clustered χ^2^ test, *P* value ≤ 0.05.

† Proportion is significantly different compared with pilot testing study using clustered χ^2^ test, *P* value ≤ 0.05.

‡ No statistical test was conducted because of small sample size.

In the 200 student survey, 95% (190) of students reported that handwashing with soap and water reduces diarrhea, 94% (189) reported washing hands before eating, and 85% (171) after toileting. However, 100% (199) noted the lack of soap and a specific place to wash hands at school (Table 5).

**Table 5:** Reported knowledge, practices, perceptions and barriers related to handwashing using closed-ended questions during the baseline formative study in urban Dhaka and rural Mymensingh, Bangladesh, 2012–2013

Indicators	*N* = 200
	% (*n*)
Perceived knowledge	
Believe that not washing hands causes diarrhea	56 (1)
Believe that not washing hands with soap and water after defecation causes diarrhea	66 (3)
Believe that not washing hands with water regularly causes diarrhea	10 (0.5)
Believe that washing hands with soap and water before eating causes less diarrhea	190 (9)
Reported handwashing practices	–
With water only	127 (6)
With water and soap	31 (1)
With water and ash	1 (0.5)
With water and mud	40 (2)
Perceived barriers of handwashing	
Lack of a specific place	199 (9)
Intermittent/unavailable water supply	59 (3)
Lack of soap	180 (9)
Most common reason for the unavailability of soap	
Lack of funds to purchase soap	107 (5)
Bar soap get used up quickly	19 (1)
School students steal soap	6 (0.3)
Teachers kept bar soap locked up	3 (0.1)
Perceived subjective and descriptive norms for handwashing	
Feeling of strong obligation to wash hands every time after defecation	156 (7)
Teachers, parents, and friends approve washing hands	142 (7)
Encouragement by teachers and friends can increase handwashing practices	187 (9)

During the in-depth interviews, rural students reported using soil to clean their hands after defecation, and urban students reported washing their hands with water only as no soap was available. Some students noted that teachers kept some soap in their room, but it was not commonly available to students.

One male student of grade 4 at an urban school said: *I think teachers will scold me if I go to collect bar soap from their room, so I wash hands with water only and dry them with my school uniform.*

One female student of grade 5 at a rural school said*: We feel bad to rub our hands with soil after toileting.*

Teachers and school management committee members noted they lacked school funds to purchase soap, and theft and misuse of bar soap were barriers to providing soap for students to wash their hands. Although the urban schools had a separate location for handwashing with piped water and a basin next to the toilet, the unavailability of soap was a barrier for handwashing. Rural schools did not have a separate location for handwashing, nor even a stored water reservoir inside the toilet. Students used a *bodna* (small jug) to collect water from the tube well or nearby pond during summer.

One female head teacher at an urban school said: *We don’t have money particularly to buy soap. Even if we provide, students play football with the soap, so I stopped providing.*

One male head teacher at a rural school said: *We were keeping soap, but the rats were taking them away frequently.*

During the participatory exercises, most of the students identified vomiting and headache as diarrheal disease symptoms, ranked comfort, disgust, and social acceptance as motivators (Supplemental Figure 2), and drew both soap and water together as basic amenities for handwashing at school compounds.

### Phase 2: Piloting the intervention for acceptability and feasibility.

The spot checks of facilities found the handwashing stations that the project had provided were functional and filled with soapy water after the intervention was implemented (Table 3).

During the structured observations, fieldworkers observed 396 students’ opportunities for handwashing: 49% (195) before eating, 47% (187) after toileting, and 4% (15) after cleaning the toilet. In the 195 handwashing opportunities before eating, 45% (87) of children washed both hands with soapy water; 55% (108) did not wash hands at all. In the 186 handwashing opportunities after toileting, 83% (155) of children washed both hands with soapy water (Table 4).

During the qualitative assessment, students who were observed to wash hands reported both physical and psychological health benefits of handwashing that provided germ-free hands, a good feeling with clean hands, and more school attendance with more opportunities to play with friends.

One female student of grade 5 at an urban school said: *I felt disgusted while I could not wash hands before the intervention. Now, every time I wash my hands with soapy water after using the school toilet, it makes me feel that my hands are clean and germ-free.*

By contrast, students who were observed not to wash hands tended to pretend that they washed hands during the interviews. Some of them believed that germs are invisible and can never be removed by washing hands.

All the students, teachers, parents, and school management committee members reported that the handwashing stations were attractive, easy to use, child friendly, and visually reminded them to wash their hands. They also mentioned that soapy water was a low-cost option of providing water and soap together. Students said they shared the idea with their family. They perceived that fixing cue cards in school compounds and viewing the pictorial flipcharts in hygiene classes were good ways to impart knowledge.

One female student of grade 5 said: *The 10 L bucket (basin) contains waste handwashing water that looks really dirty. We never saw it when we washed our hands before, and the wastewater drained away. Now we can understand how dirty our hands were.*

One male student of grade 5 at a rural school said: *Now we have all the things (water and soap) in one place: just have to pump the bottle to get soapy water and turn on the tap to get water from the bucket to wash hands.*

One female school management committee at a rural school said: *Actually, it is a good initiative (the intervention) to keep the children clean and hygienic.*

Teachers and management committee members of the hygiene committee formed a fund at each school and reported that they decided to contribute US$0.25 to 1.25 each month for purchasing low-cost detergent powder (US$0.75) per sachet for daily use or 1-kg pouch bags for longer use. Since the detergent powder was low cost, the head teachers at each school mainly covered such costs from contingency funds and/or their own pockets. They also mentioned that the formation of hygiene committee institutionalized the intervention by encouraging all members to contribute to detergent costs, encouraging the formation of habitual handwashing, and disseminating information to the community.

One head teacher at a rural school said: *Every work needs to be done through a system, and hygiene committee is such a good system to maintain the promoted activities at school compound.*

One head teacher an urban school said: *Soapy water requires such minimal amount for which we all (teachers and school management committee members) are paying and can continue funds in future as well.*

Maintenance duties for student members of the hygiene committee at three schools and the cleaning staff at one urban school included storing water, preparing soapy water and cleaning all the handwashing stations. Students perceived the pump top soapy water bottle as similar to using liquid soap. However, sometimes the pump became stuck, causing rural school students to miss classes trying to repair the bottles.

One male student of grade 5 from the hygiene committee at a rural school said: *We start the school day by cleaning, refilling water, and preparing soapy water; otherwise, students will not be able to wash hands.*

### Phase 3: Follow-up assessment at 14-months to explore sustainability.

During the spot checks of facilities, fieldworkers identified that one urban school stopped using the soapy water bottle, but the remaining schools continued to use them (Table 3).

During the structured observations, fieldworkers observed 1,055 students’ opportunities for handwashing: 56% (586) before eating, 44% (465) after toileting, and 3% (4) after cleaning the toilet. In the 586 before eating events, 9% (55) washed both hands with soapy water; 89% (520) did not wash hands at all. In the 465 after toileting events, 37% (172) washed both hands with soapy water; 38% (175) did not wash hands at all (Table 4).

During the qualitative assessment, students who were observed to wash hands reported that handwashing habits with soapy water resulted in fewer diarrheal disease episodes that motivated them to wash their hands, and they disseminated the idea of preparing soapy water to their family members, neighbors and relatives.

One male member of parent–teacher association at a rural school said: *My daughter is at grade 4. She told me to arrange soapy water at home for handwashing as it was provided at school. So, I brought liquid soap for her from the market because I don’t have the pump or the bottle to prepare soapy water.*

Students who were observed not to wash hands, especially in urban schools, reported that they did not wash their hands as they were in hurry to play and the lack of a handwashing station with soapy water demotivated them.

Teachers, management committee members and janitors at urban schools commonly mentioned shortages of teachers and janitors, increased workload and inactive management committee members who were reluctant to maintain the handwashing stations and to contribute to the recurrent cost of detergent.

One female janitor at an urban school said: *Sometimes I prepare soapy water and sometimes I ignore because male students mostly play with the soapy water bottle. They make bubbles with soapy water, spread it all over the toilet and throw the bottle to other students. This way they finish it. So, I don't want to refill it though teachers told me to do, but how many times I should refill the bucket and the bottle? It becomes difficult for me to clean the toilet and hardware. So, I stored all hardware inside.*

One female head teacher at an urban school said: *The president of the school management committees is sick, vice president is outside of Bangladesh, and one teacher has been resigned from this school. Our janitor, who was proactive, also died last month and students of grade 5 have finished their studies and left for high schools. Therefore, the hygiene committee became inactive to continue handwashing activities in the school.*

Rural schoolteachers, management committee members, and janitors noted that perceived health benefits, active hygiene committee supervision and involvement of students in the maintenance activity had played a vital role in continued use of the handwashing stations and contributions to the recurring costs of detergent.

One male student of grade 5 at a rural school said: *Our head teacher always ensured the handwashing facilities were available at school compound. He distributed the overall maintenance responsibilities of the handwashing station to the students of the hygiene committee, so they refilled water in the bucket, prepared soapy water and kept the station clean.*

## DISCUSSION

Our baseline data suggested handwashing with soap was not a common practice among school children in Bangladesh, and almost nonexistent before eating and after toileting. Previous studies suggest that barriers to sustained behavior change of WASH interventions include high cost of products, lack of infrastructure, habit formation, motivation, and maintenance.^33^^,^^39^^–^^41^ This pilot intervention attempted to address these barriers.

Our study showed that schools found soapy water a low-cost (US$0.05 per day for daily handwashing in the school) and child-friendly product that created access and opportunities for handwashing practices. The school community commonly credited the increase in handwashing rates to this low-cost hand cleaning agent that transcended the common barriers of bar soap related to high cost (the average price of common bar soap is US$0.35), theft, and misuse of bar soap in the school.^13^^,^^42^

The lack of infrastructure (availability of running water, basin, and soap) to promote handwashing at key times is common in schools in low-income countries including Bangladesh^24^^,^^43^ and most of this study’s schools. Therefore, soapy water was dispensed as part of the handwashing stations in this study. These handwashing stations provided a complete package of convenient infrastructure and worked as a structural and enabling factor to influence handwashing behavior change.^10^^,^^44^^,^^45^

Students and teachers indicated that the promotion of handwashing with soapy water was integrated into the school’s curriculum using weekly sessions and exams. This suggests that the intervention supported individual psychosocial factors of habit formation related messaging (e.g., key times for handwashing) and establishment of handwashing habits and development of cues or nudges among students that trigger and reinforce habits such as easily accessed handwashing station and visual reminders (e.g., cue cards and hygiene committee). Students also identified perceived health benefits and concerns about disgust and cleanliness as sustained affective motivations for handwashing with soapy water.

Marteen et al. suggested that successful sustained adoption includes 1) the behavior, 2) the frequency of the behavior practice, and 3) the length of time at which this behavior should be measured to be considered sustained.^33^ This intervention achieved sustained adoption. Handwashing with soapy water significantly increased after 30 days of intervention at two key times: before eating (0 to 46%, *P* < 0.05) and after toileting (0.7–82%, *P* < 0.05), that emphasizes the importance of providing essential facilities.^46^^–^^48^ Although handwashing behaviors did decline over time, handwashing after toileting with soapy water (0.7% versus 37%, *P* value ≤ 0.05), and handwashing with soapy water before eating (0% versus 9.4%, *P* value ≤ 0.05) were still much higher at 14 month than at baseline, suggesting some level of habit adoption. The spot checks and structured observations data from 14 months follow-up also suggest that this handwashing intervention has some potential for sustainability as three of the four schools maintained the handwashing stations stocked with soapy water. This finding contrasts with Kenya and India where schools did not maintain soap at handwashing locations when assessed within a few months or years postintervention.^49^^,^^50^ Additionally, teachers and school management committee members in this study continued covering the recurrent cost of detergent even after the project ended in three of the four schools, observed at 14 months follow up. Personal attitudes and behaviors of the school leadership may have directly influenced maintenance and behaviors.^51^^–^^53^

However, the lack of maintenance a handwashing habit over time might have been affected by the cessation of weekly hygiene sessions (Table 3), inactive management committee members and lack of ongoing availability of essential supplies. Education and provision of materials may be insufficient to ensure long-term adoption of habitual handwashing. Therefore, the long-term success of a school WASH intervention requires a system of school funding with standardized roles and responsibilities for maintenance.^54^ In this pilot intervention, engagement of school communities to form hygiene committees was effective to support operations and maintenance, create champions and promote ownership. It was effective in convincing teachers and school management committee members to cover the recurrent cost of detergent powder for soapy water preparation from the schools’ contingency funds (US$9 per month),^25^ janitors and students ensured maintenance, and students and teachers monitored and motivated handwashing behaviors among students and peers. Having a system in place to cover recurrent costs has the potential for increased intervention sustainability in settings with limited resources. One study conducted in Bangladeshi schools also identified recurrent financial support for operations and maintenance by the government or community, a maintenance plan and a champion (possibly teachers) as important conditions for continued management of school sanitation.^25^ In other contexts, community involvement and support improved accountability of a routine immunization system in Nigeria,^55^ in reproductive health outcomes in Malawi,^56^ in public service delivery in India, in detecting maternal deaths in Malawi,^57^ and in funding school sanitation in Bangladesh and Belize.^25^^,^^58^ However, a study in Kenya schools found that funding remained a major barrier for sustainable allocation of recurrent costs for soapy water provisions.^14^

The Ministry of Primary and Mass Education increased the school WASH allocation from US$36.5 million (BDT 2918 million) in 2016–2017 to US$57.4 million (BDT 4591 million) in 2017–18 financial years for school WASH.^59^ The budget includes financial provision of drinking water supply and sufficient WASH blocks. Each WASH block consists of three toilets, two urinals and hand and foot wash facilities constructed by the Department of Public Health and Engineering (DPHE) through the primary education development project and supported by development partners.

The Ministry of Education allocates up to US$240– US$370 per school per year under the School Level Improvement Program fund for overall maintenance and school improvements^25^^,^^60^ and recommends school management committee and parent teacher associations provide US$125– US$625 (BDT 10,000–50,000) (personal communication with the Deputy Director of Planning at Primary Education). Schools can use this recommendation and engage active community members to overcome the weak performance, increase accountability and champion the continued management of school handwashing.

In research among 117 Bangladeshi schools applying a Life Cycle Costs Approach (LCCA),^61^^,^^62^ a methodology to estimate future costs, IRC WASH, a nonprofit organization supporting government WASH interventions, estimated US$10 (BDT 814) per student is needed to construct water and sanitation facilities in schools, and US$1.40 (BDT 108) per student per year is needed for recurrent costs of supplies and maintenance of equipment.^43^ Taking the average size of the student body at Bangladeshi schools (700) from a nationally representative survey,^28^ we calculated total US$395 per school per year is needed particularly for such recurrent costs. However, US$240–370 allocated under the School Level Improvement Program, is meant to cover several school and educational improvement related expenses and is not specifically allocated for WASH maintenance.

Though the fourth Primary Education Development Program estimated US$10.5 million as overall recurrent expenditure for financial years 2019–2023 (that included administration/the salaries for existing teachers and personnel and other recurrent costs), maintenance and supplies for WASH costs are not mentioned.^60^

The Ministry of Education should provide sufficient WASH supplies and include preventive measures linked to handwashing to prevent the rapid spread of communicable diseases like COVID-19 through public schools in Bangladesh. The World Health Organization recommended a soapy water solution, similar to what we deployed in this study, as an alternative hand cleanser to help interrupt the transmission of COVID-19.^63^ Soapy water has been found to be effective as bar soap in removing indicator organisms from hands and is more effective than water alone.^42^ As schools are the potential places for pathogen transmission the feasible and low-cost approaches identified from this study could be promising.

There are some limitations to the conclusions that we can draw from this study. We included only eight schools, though they represented a typical and similar environment to other schools in Bangladesh. The short-term intervention (30 days) might have impacted longer term habit adoption, implying the need for further efforts to improve long-term behavior change. Structured observations by fieldworkers likely altered handwashing practices, though this is a less biased approach to assess hygiene behaviors compared with self-reporting.^64^^,^^65^ However, the majority of student did not wash hands with soapy water, hence that presence of an observer likely did not completely alter the targeted practices. This study was conducted in 2012. However, the more recent national hygiene survey conducted in 2018 showed that only 39% of the school boys and girls had improved, unlocked, accessible toilets that have soap and water available.^66^ Hence the constraints identified in our 2012 study have persisted in Bangladeshi schools.

This study piloted an innovative combination of appropriate technology, knowledge, and skill acquisition interventions to promote handwashing in a school setting. The training of students with targeted communication materials and formation of hygiene committees sought to make handwashing with soap at two key times a sustained change in behavior. Although direct government support for the supplies necessary to maintain handwashing were insufficient, following the intervention hygiene, committees in several schools were able to restock supplies and even 14 months later support some improved practices. Efforts to improve government support for these essential public health supplies should continue, but, in the interim, low-cost infrastructure and hygiene committees can contribute to a safer educational environment and safer society.

## Supplemental tables


Supplemental materials
