# Supplementary material for: Pilot of a Low-Cost Elementary School Handwashing Intervention in Bangladesh: Acceptability, Feasibility, and Potential for Sustainability
Source: Am J Trop Med Hyg. 2021 Nov 29;106(1):239–49. doi: 10.4269/ajtmh.20-1335 (PMC8733513; doi:10.4269/ajtmh.20-1335)
Supplement: Supplementary file 1 [file tpmd201335.SD1.pdf]

Supplemental Figure 1: Cue-cards depicting handwashing practices using two handwashing stations with soapy water at recommended key times

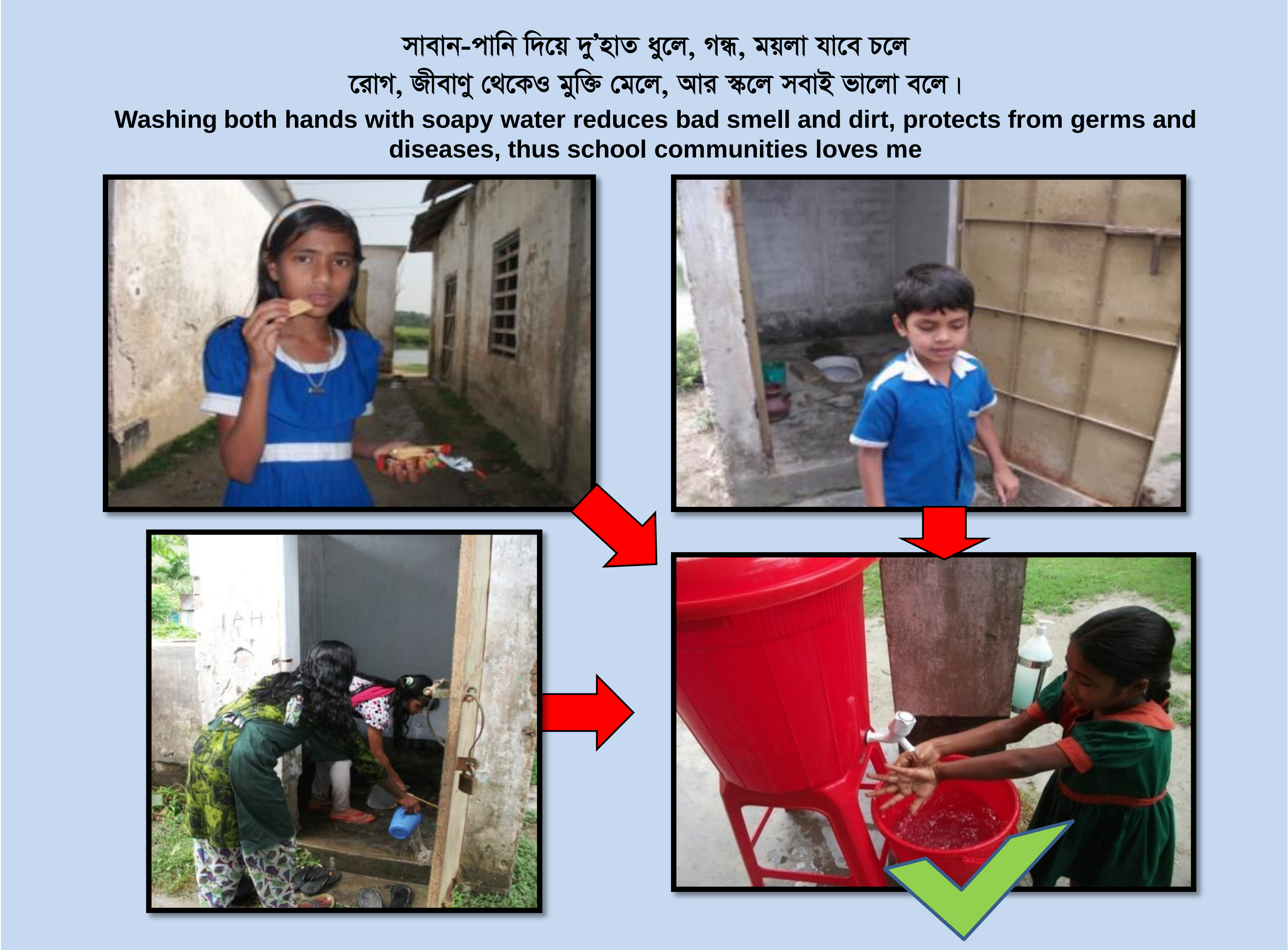

Photo credit: Mahadi Hasan

Supplemental Figure 2: Customized handwashing motivators displayed to the formative study schools during the participatory exercises

Social acceptance	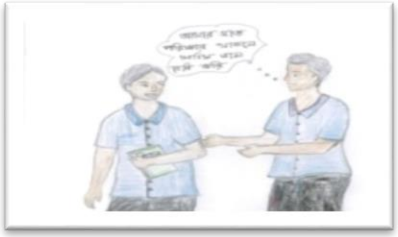
Disgust	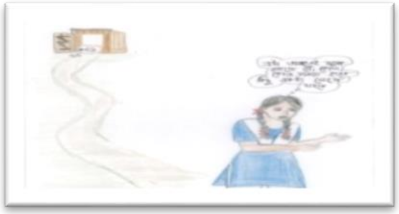
Comfort	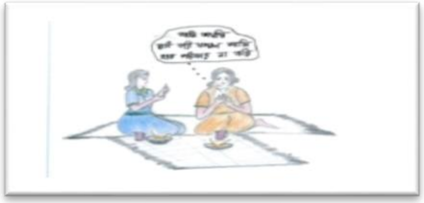

Illustration credit: Farhana Sultana

Supplemental Table 1: Phases of piloting hand hygiene practices in schools in urban Dhaka and rural Mymensingh, Bangladesh 2011-2013 study phases and activities

Phases in the study	Quantitative methods and #	Qualitative methods and #	Implementation of intervention
**Phase 1**<br>Formative study	Baseline data collection<br>• Spot checks in school facilities (4)<br>• Structured observation of students (12)<br>• Student surveys (200)<br>Intervention development<br>• Structured interview of teachers (8) and students (100)	• In-depth interviews of head, science and assistant teachers, and janitors (16)<br>• Focus group discussion of school management committee members, male and female students (12)<br>• Participatory exercises of students (12)	Implementation, pretesting and trial of improved practices of hand hygiene behavior
**Phase 2**<br>Piloting the intervention	• Spot checks in school facilities (4)<br>• Structured observations of students (24)	• In-depth interviews of janitors (2)<br>• Focus group discussions of students, teachers, management and hygiene committee members (14)	• Training of teachers to deliver hand hygiene sessions<br>• Provision of two handwashing stations<br>• Provision of flipcharts and cue cards<br>• Formation of hygiene committees
**Phase 3**<br>14-month follow-up assessment	• Spot checks in school facilities (4)<br>• Structured observation of students (12)	• Focus group discussions of male, female students and teachers and management committee members (12)	

Supplemental Table 2: Handwashing station used in the study intervention

Photo	Name (Capacity)	Cost	Description
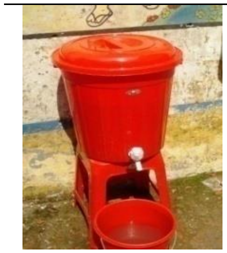	1. RFL* bucket (40 L), Stool and small bucket (10 L)	US$5	The handwashing station consists of a plastic bucket with a lid, a plastic turn-handle spout, and a plastic stool for the bucket to stand on. We also provided a small plastic bucket for rinse water collection.
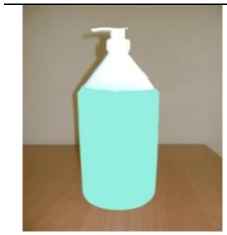	2. Soapy water bottle with pump (1.5 L)	US$0.6	A translucent 1.5 liter plastic pump topped bottle (combination of design # 1 and 3) Single-use 30g packets of detergent powder were mixed with water to make "soapy water "for handwashing.
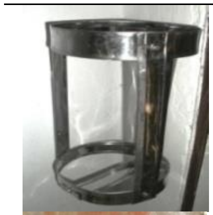 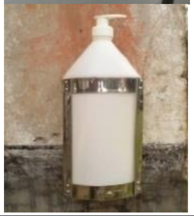	3. Holder	US$5	The holder uses a steel bar to attach it to the wall and to hold the soapy water bottle (design # 2).

- RFL = Rangpur Foundry Ltd , a professionally managed, ISO 9001 certified organization in Bangladesh that serves the global needs forcastiron, PVC and plastic products.

Supplemental Appendix 1a: Physical Survey/ Spot check of facilities at baseline

Objective:

What are the factors in the physical environment favoring or discouraging hand washing with soap after toileting or after a respiratory related condition/ before eating food?

Instruction:

- This exercise will be carried out by 1 researcher to do a spot check of the school facilities
- Introduction with the head master will be done and the letter of permission from the Department of Education will be shown
- Tell them that you are conducting a study on child health in Bangladesh, and that is why you are here
- Tell them that you would like to look at their school facilities and that it will take 30-40 minutes
- In consultation with the head teacher and teachers decide on who will tour you of the school facilities

Questions and code		
1.1	ID number of school	☐☐☐☐
1.2	SHEWA B Cluster number	☐☐☐
1.3	District name and code<br>……………………………………………………………	☐☐☐
1.4	Upazilla name and code<br>……………………………………………………………	☐☐☐
1.5	Union name<br>………………………………………………………………………………………..	
1.6	Village name]<br>………………………………………………………………………………………..	
1.7	Type of school<br>Government primary school...................................01<br>Registered non government primary school……...02<br>Madrasha..03<br>Technical school...04<br>Other(specify)...99	☐☐
1.8	How many shifts are there?	☐☐
1.9	Identification number of observer	☐☐
1.10	Date of visit (DD/MM/YY)	☐☐/☐☐/☐☐
1.11	Start time (Hour: Minute)	☐☐/☐☐

TOILETS:

2.1	Are there any toilet facilities in the school? (Ask and check)]<br>Yes.........................1<br>No..........................2 (*If answer is no, skip to 2.11)*	☐
2.2	How many toilet facilities (latrine stalls) are there in total? (Ask and check)	☐
2.3	How many latrines are there for separate use:	☐<br>☐

	a.______ [# latrines for girls]<br>b.______ [# latrines for boys]<br>c. .... [# latrines for boys and girls both]<br>d.______ [# latrines for girls and female teachers]<br>e.______ [# latrines for boys and male teachers]<br>f. _______[# latrines for teachers only]<br>g._______ [for all]	
2.4	What is the distance of the closest latrine from the classrooms?<br>1. Within 3 feet<br>2. Outside three feet but within 10 feet<br>3. Beyond 10 feet	☐
2.41	Is this latrine functional?<br>1. Yes<br>2. No	
2.5	Where do children most commonly wash their hands after coming from the toilet?<br>1. Inside the toilet<br>2. Outside of latrine but within 3 feet of latrine<br>3. No specific place<br>4. At the water source (tube well)<br>5. Others ________________________	☐
2.6	Is the latrine usable throughout the year? (Ask only)<br><br>All year round..01<br>Dry season only..02<br>Wet season only..03<br>Other (specify)....................................99	Type of latrine Code<br><br>1.<br>2. ☐☐<br>3.<br>4.<br>5.
2.7	In general what is the state of cleanliness of the school toilets?<br>Clean<br>1. Slightly dirty]<br>3. Very Dirty] (Visible stool, dirt on the slab and floor)	☐
2.8	Are the toilets well lit?[Is there a light inside the toilet]<br><br>1. Yes<br>2. No	☐
2.81	Are the toilets well ventilated? [Space inside walls/ metal grates used for the free flow of air]<br><br>1. Yes<br>2. No	
2.9	Is there strong odor / smell inside the toilet?<br><br>1. Yes<br>2. No	☐
2.10	Is there a specific person assigned for cleaning the toilets and water source? (toilet, tube well area, washing/hand washing area)<br><br>1. Yes	☐

	2. No	
2.11	If there are no toilet facilities, where do students go to urinate and/or defecate?(Ask only)<br><br>Open...01<br><br>In nearby facility/ toilet]..02 → Skip to *2.13*<br><br>Other (specify)].............99 → Skip to *2.13*<br>______________________________<br>______________________________<br>______________________________<br><br>If answer to 2.11 is 2 or 99 go to Question no 2.13	☐☐
2.12	If open defecation, location of open defecation site (Ask and check)<br><br>Within the school premises...................................01<br><br>Nearby bushes but outside the school premises...02<br><br>[Other specify)] 99	☐☐
2.13	Are there visible faeces on and around the school grounds? (Do not ask. Observe only)]<br><br>Yes..........................1<br><br>No...........................2	☐
2.14	Is there any smell of faeces in and around the surrounding area of the school? (Do not ask. Check only)]<br><br>Yes.........................1<br><br>No...........................2	☐

3.1<br><br>Latrine<br><br>#	3.2<br><br>Latrine type<br><br>(See code from below)	3.3<br><br>[Latrine User]<br><br>[Girl's latrine]..............1<br>[Boy's latrine]..............2<br>[Teacher's latrine]........3<br>[Non specific]...................4<br>[Other(Specify)]........77<br><br>(See if the door of the latrine mentions the target user . Confirm by asking the students/ teacher who uses the specific latrine)	3.5<br><br>Hand washing location<br><br>1. Inside the toilet<br>2. Outside the toilet<br>3. Near tube well<br>4. Corridor<br>Others (specify)	3.4<br><br>Did the latrine user wash his/her hand?<br><br>1. Yes<br>2. No<br>3. Unable to observe<br>(if answer 2 or 3, then end of the observation)	3.6<br><br>[Did they use any anal cleansing materials available in the toilet facilities?]<br><br>[Toilet paper]..................1<br>Cloth].........2<br>Mud ....3<br>Nothing present ....4<br>(Leaves)..5<br>[Other(Specify)]......77<br>(Reported)	3.7<br><br>[Is there soap available at the HW location?]<br><br>[Yes]...1<br>[No].....2<br><br>(Observe only)	3.8<br><br>If soap is present at this handwashing location, does it look as if it has been recently used?<br><br>Yes...1<br>No....2	3.10<br><br>Hand drying technique<br><br>1. Air dried<br>2. Did not dry<br>3. On their apparel<br>4. Towel(common)<br>5. Individual towel<br>6. tissue paper<br>7. Others (specify)	Comments

3.2 Code for Question 3.2

Toilet Facility Type

Flush or pour flush toilet flushed to:

Piped sewer system... 01

Septic tank.. 02

Flush to pit latrine (Offset)...03

[Pit latrine with slab & water seal]... 04

[Pit latrine with slab & no water seal but with a lid] 05

Ventilated Improved Pit (VIP) latrine] 07

[Pit latrine with slab and flap, no water seal] ………………………...08

[Composting toilet, *(Composting toilet ensure separation of urine, water and excreta)*] 09

[Flush or pour flush toilet connected to somewhere else (canal, ditch, river, etc.)] 10

[Pit latrine without slab/open pit]..11

[Pit latrine with slab & no water seal/broken water seal and no lid] 12

[Hanging toilet/latrine]... 13

(Open defecation):

[No facility/bush/field]... 14

[Others: Specify]... 77

Not Applicable..99

Section 4: <br>***Water source***		
4.1	Is there a water source in the school? (Ask only)<br><br>Yes...........................1	☐

	No...........................2 [Skip to 4.8]	
4.2	How many water sources? (Ask and observe)	☐
4.3	What is the source of the water? (Ask and Observe)<br><br>Shallow tube well. …..01<br><br>Deep tube well]. ………………..............................02<br><br>Protected ring/dug well……………...........................03<br><br>Unprotected dug well………...............................……04<br><br>Tara pump..05<br><br>Arsenic free treatment plant ....06<br><br>Water from protected spring…...............................07<br><br>Water from unprotected spring.......................................08<br><br><u>Surface water:</u><br><br>Rainwater…………<br>…………………………………….……...........09<br><br>Tanker truck………………………….................10<br><br>Cart with small tank…………….............................11<br><br><u>[Pathogen treatment plant (Pond Sand Filter)]:</u><br>River/dam/lake/ponds/stream/canal/irrigation channel.]...12<br><br>**[**Directly from River/dam/lake/ponds/stream/canal/irrigation channel]…....13<br><br>Piped water into the school]…..14<br><br>Public tap/stand pipe]..15<br><br>Other: [specify]..77	a. ______<br><br>b. ______<br><br>c. ______
4.4	Is water available all year round? (Ask only)<br><br>All year round]..1<br><br>[Dry season only]..2<br><br>[Wet season only]..3	a. ______<br><br>b. ______<br><br>c.______

	[Other (specify)]...77	
4.5	How far is the nearest water source from the classroom?<br><br>1. Within 3 feet<br>2. Outside three feet but within 10 feet<br>3. Outside 10 feet	☐
4.51	Does the nearest water source have water for drinking all year round?<br><br>1. Yes<br>2. No	
4.6	Who cares for or maintains (washing surrounding area and cleaning) the water point?<br><br>(Ask only)<br><br>[Students]..1<br><br>Student brigade].....................................2<br><br>School staff] ....3<br><br>Hired person /janitor]..............................4<br><br>Other: specify]............................ 77	☐☐
4.7	Did the source of water point observed look clean? [Considering presence of cow dung, solid waste etc.] (Observe only)<br><br>Yes........................1<br><br>No..........................2<br><br>Don't know..........99	a. ______<br><br>b. ________<br><br>c. _________
4.71	How many hand washing points are there?	
4.72	How far is the nearest hand washing point from the classroom?<br><br>1. Inside the classroom<br>2. Outside classroom but within 3 feet<br>3. Outside of the classroom but within 10 feet<br>4. More than 10 feet away<br>5. Others ________________________	
4.73	Is this hand washing station functional?<br><br>1. Yes<br>2. No	

4.8	What device is used for water supply for this hand washing station? (Observe only)<br><br>[Same as Question 4.3]....................................1<br><br>[Specially designed hand washing station (A drum with a tap)]..................2<br><br>[Tap ....3<br><br>[Water container (e.g. bucket, Bodna)].....4<br><br>[Other: specify]..77	☐☐
4.9	Is there water in the device mentioned in q4.8? (Observe only)<br><br>Yes.........................1<br><br>No...........................2<br><br>[Don't know]............99	☐☐
4.10	If it is a hand washing station, how often is the water added to in the hand washing station? (Ask only)<br><br>Daily..1<br><br>1-2 days...2<br><br>3-4 days...3<br><br>Never..4<br><br>N/A..5<br><br>Other: specify.................................... 77	☐☐
4.11	Are there hand cleansing agents available near the hand washing station? (Observe only)<br><br>Yes........................1<br><br>No...........................2 **[**skip to 4.14]	☐
4.12	What types of hand cleansing agents are available? (Observe only)<br><br>Soap..1<br><br>Detergent.....................................2 [Skip to 4.14]	☐☐

	Q [Ash]..3 [Skip to 4.14]<br>[Mud]...4[Skip to 4.14]<br>Nothing …………………………….. 5<br>Other: specify ....77 [Skip to 4.14]	
4.13	Where is the soap for hand washing usually located? (Ask to see the location of the soap, and fill out response according to observation)<br>Next to hand washing station..................................1<br>Away from Handwashing station<br>Next to or outside the toilet..2<br>In the teachers room/office room.................................3<br>In the classroom..4<br>Inside the toilet..5<br>[Other: specify]..77	☐☐
4.14	Are there any additional supplies of soap stored at school for hand washing?(Ask and check before writing the response)]<br>Yes].........................1<br>No]...........................2<br>Don’t know].............99	☐☐
4.15	Do you have any additional places or items (e.g. bucket, basin or container) to wash hands in?] (Ask and check)<br>[Yes].........................1<br>[No]...........................2 [stop here]<br>[Don’t know].............99 [stop here]	If yes, go on to 4.16<br>If no, stop ☐☐
4.16	[Is there water in the device asked about in<br>q 4.15?](Observe only)<br>[Yes].........................1<br>[No]...........................2	☐☐

	[Don't know].............99	
4.17	If this is a hand washing station, how often is the water added to in the hand washing station?(Ask only)<br><br>[Daily]..1<br><br>[1-2 days]..2<br><br>[3-4 days]..3<br><br>[Never]..4<br><br>[N/A]...5<br><br>[Other: specify]............................ 77	☐☐
4.18	Are there hand cleansing agents available near the hand washing station?<br><br>[Yes].........................1<br><br>[No]...........................2 [stop here]<br><br>[Don't know].............99 [stop here]	☐☐
4.19	What types of hand cleansing agents are available?](Observe only)<br><br>[Soap]...1<br><br>[Detergent]....................................2<br><br>[Ash]...3<br><br>[Mud]...4<br><br>[Other: specify] 77	☐☐

Supplemental Appendix 1b: Physical Survey/ Spot check of facilities after the intervention

**[**DESCRIPTION OF SCHOOL**]**	
[NAME OF SCHOOL]	
[ID NUMBER OF SCHOOL]	

**[**FOLLOW-UP RELATED INFORMATION**]**	
[DATE OF FOLLOW-UP]	
[NUMBER OF FOLLOW-UP]	
[NAME OF HWS]	
[DATE OF INSTALLMENT]	

[DESCRIPTION OF INFORMANT]

[NAME OF RESPONDENT]	
[SEX OF RESPONDENT]	
[AGE OF RESPONDENT]	
[ACADEMIC QUALIFICATION]	
[OCCUPATION OF RESPONDENT]	

Introductory statement:

We are from **icddr, b.** We are coming to hear about the experiences you have had with the handwashing station since we installed it here. We want to learn what you like about it, and what you don't like about it.]

- **[**Seek the consent of the participant to enter inside the office/classroom and make the visit]

SECTION: (Immediate Reaction & General Information about the handwashing station/HWS)

[Initial reactions]

1. Write any questions or reactions the householder has during the initial greetings, or after hearing that you are coming to ask questions about the handwashing station]

2. When do people wash their hands, before or after which activities, or at which time of day? Write or use the table if preferred]

**[Events:]**	**[HWS used]**	**[Time of the day]**
1- After defecation		

2- Before eating		
3- After cleaning school latrine		
4- Other (Specify)		

Additional comments:

3. Ask who in the household uses the handwashing station the most, and why?]

4. Are the children capable to use it? Are they performing hand wash themselves? How the parents are assisting their children? Is the HWS within their (children) reach to use it?]

5. How did the teacher/SMC-PTA members/students/your friends react to this hand washing station? (Were the interested? Did they want one for their own? Did they inquire about how you got it? Did they make any comments regarding its use?]

[General Information about Hand Washing Station]

6. What do you like about the hand washing station? Ask separately for each HWS if applicable. (Possible answers/probes: HW station was provided free of charge, serves as a reminder to wash hands? Soap and water ready together when it is needed? Easy for the children to use, convenient location? For aspects mentioned, follow up for details, (why it is convenient? How do children find it more convenient? How it is so compared to their previous hand washing station?]

7. How did you like the soap water? Did you like the feel/smell/overall experience of washing your hands with it? Did you have to remake the soapy water yet? Describe how you did it (issues/ease)

Section: B [Problem facing with Hand Washing Station]

8. What problems you had with the hand washing station? (Probes: Need to cope, HWS placement problem, preparing soapy water is a problem, maintenance/refilling issues] For issues mentioned, follow up for details and ask why this is so and how it can be resolved?]

9. Which part of the HWS you don’t like? What parts of the HWS are not liked by the teachers, hygiene committee, cleaning staff or other students of the school? What do they like or do not like about the HWS?]

10. How do you refill the hand washing station? Are there any problems/issues regarding the refilling of the HWS? [**Please note them clearly, if applicable, so that they can be addressed in the next follow up visit**.]

HWS :
Soapy water bottle:

11. Does the hand washing station moved after installation? Would another location be more convenient? Why or why not?)

SECTION: C (SUGGESTION)

12. What problems they you had with the hand washing station? For issues mentioned, follow up for details and ask how it can be resolved?)
13. How can we change that part of the HWS which you do not?)
14. How we can increase the use of handwashing station among entire school children?
15. Additional comments

Section D: (Participant/Focused observation)

(Place yourself in a convenient space from where you can observe HWWS in each one hour)
Time start ____________ am /pm End time __________ am/pm

16. (List the use of HWS)

Prompts 1. Who is involved in water collection and soapy water preparation (responsible person, teacher, hygiene committee members) 2. What are the sources of water 3. Who are the users 4. Usage of soapy water/water/both 5. Body language, 6. Related critical event, 7. Notes on usage
Event 1:<br><br>Event 2 :<br><br>Event 3 :

17. (Please note the person, name, roll and class of the student if they did not wash hands before eating, after toileting and after cleaning toilet. Later discuss the reason behind not washing hands and not using the HWS.)

Event 1 :<br><br>Event 2 :<br><br>Event 3 :

1. Is anything broken? (1=Yes, 2=No) Note what_________ ____________________

2. Is any part missing? (1=Yes, 2=No) Note what________

3. Is there water in it? (1=Yes, 2=No)

4. Is there soapy water? (1=Yes, 2=No)

5. Is the device functional? (1=Yes, 2=No)

6. Has this HWS been moved from the initial place of installation? (1=Yes, 2=No)

7. What evidence makes you think that the device is in use? ___________ ____________

8. What evidence makes 8.you think that the device is not use? ______ _________________

9. Note any other problem that you see, which needs to be fixed (such as flooding, or any other) ________

19. [Please fill the first column with the problems you have observed/noticed regarding the hand washing station and its use. Then discuss these problems with the respondents and ask them to offer a solution. If they are unable to do so, suggest one and come to an agreement regarding the solution to the problem.]

Problems/Issues observed)	Solution agreed upon)<br><br>(Note who suggested the solution/whether no solution was reached)	(Resolved Status)

Please repeat the issues and the proposed solutions before you leave the household. Please bring a copy of this table in the follow up visit to this household to see if it was resolved

20. Below mentioned information are to be collected through discussions among the students of grade IV and V)

(School ID): (Name of the interviewer):

(Total no. of students at class IV and V): (Total no. of students present at the FGD):

- (Ask students if they are using the equipment? Do they use the HWS? Why or why not?)

- (Did they observe anything new or any change about the HWS? What is their perception after using the HWS? How was the HWS?)

- **What things they liked? Stores water, innovativeness etc)**

- **(Why they did not like the HWS? (Water becomes finished, friends influence in not using etc)**

21. **(What type of problems they faced in using the HWS? Talk to the head teacher, MLSS or another responsible person)**

**(Hardware)**	**(Type of problem)**	**(Did the participants solve? (Yes/No)**	**(How)**
(40L bucket and tap)			
(Soapy water)			
(Soapy water bottle and steel holder)			
(10L bucket)			
(Flipchart, poster, cue card)			

22. Ask the members of hygiene committee/students that who is involved in maintaining the HWS? Please note all the positive or negative things that they spoke about, especially about any problem that they faced and the process of managing problems.)

23. Students recommendations: What kind of change and inclusion would motivate them in using the HWS?)

(40L bucket and tap)	

(Soapy water)	
(Soapy water bottle and steel holder)	
(10L bucket)	
(Others)	

19. Teachers recommendations: What kind of change and inclusion would motivate them in using the HWS?)

(40L bucket and tap)	
(Soapy water)	
(Soapy water bottle and steel holder)	
(10L bucket)	
(Others)	

20. H ow you will continue using the HWS, refilling water in the bucket and preparing soapy water after we left? Do you think the hygiene committee is plying an effective role in this regard? How to make the hygiene committee more effective? What is your recommendation towards the sustainability of promoting HWS in elementary schools of Bangladesh?
